# A WHOLE-HEALTH MODEL OF CARE TO PROMOTE SOCIAL AND RACIAL JUSTICE

**DOI:** 10.1093/geroni/igad104.1399

**Published:** 2023-12-21

**Authors:** Anna Faul, Sam Cotton, Pamela Yankeelov, Barbara Gordon

**Affiliations:** University of Louisville, Louisville, Kentucky, United States; University of Louisville, Louisville, Kentucky, United States; University of Louisville, Louisville, Kentucky, United States; University of Louisville, Louisville, Kentucky, United States

## Abstract

Whole health models of care aim to coordinate care across sectors, address the needs of older adults with multiple chronic conditions, and promote health equity. The FlourishCare team will present data on the effectiveness of a whole health model, the FlourishCare Model (FCM), serving 159 older adults with multiple chronic conditions, using a two-level longitudinal design and hierarchical linear modeling to test a multilevel growth model. The results showed significant growth in total FlourishCare Index (FCI) scores over time for individual health behaviors, healthcare access, and social determinants. Biological determinants showed significant growth only for rural patients. The FCM was able to support patients with lower education attainment to improve at a higher rate than those with higher education attainment, both for the total score on the FCI and for healthcare access. The FCM was also able to support rural patients at a higher rate than urban patients to gain access to healthcare, and it supported Hispanic patients the most in improving their social determinants of health. The study demonstrates the importance of a whole health approach to care, which focuses on what matters most to patients, particularly older adults who place importance on quality-of-life outcomes. The study also shows how a whole health approach can address health equity by accelerating the flourishing of rural, Hispanic patients with lower educational attainment, promoting social and racial justice.

